# Beyond the bowel: Wernicke's encephalopathy as a neurological complication of Crohn's disease: A case report

**DOI:** 10.1016/j.radcr.2024.03.088

**Published:** 2024-05-04

**Authors:** Giovanni Failla, Francesco Tiralongo, Pina Crimi, Salvatore Lo Fermo, Pietro Valerio Foti, Emanuele David, Corrado Ini, Monica Palermo, Daniele Falsaperla, Stefano Palmucci, Antonio Basile

**Affiliations:** a Radiology Unit 1, Department of Medical Surgical Sciences and Advanced Technologies “GF Ingrassia”, University Hospital Policlinico “G. Rodolico-San Marco”, University of Catania, 95123 Catania, Italy; b Neurologic Unit, AOU “Policlinico-San Marco”, Department of Medical, Surgical Sciences and Advanced Technologies, GF Ingrassia, University of Catania, Via Santa Sofia n.78, 95100, Catania, Sicily, Italy; c Department “GF Ingrassia”, Section of Neurosciences, Neurology Clinic, University of Catania, Operative Unit of Multiple Sclerosis, University-Hospital G. Rodolico - San Marco, Catania, Italy, Catania 9126, Italy; d UOSD I.P.T.R.A., Department of Medical Surgical Sciences and Advanced Technologies “GF Ingrassia”, University of Catania, University Hospital Policlinico “G. Rodolico-San Marco”, Catania, Italy

**Keywords:** Encephalopathy, Wernicke, Crohn disease, Magnetic resonance imaging, Computed tomography, Malnutrition

## Abstract

Wernicke encephalopathy (WE) is a rare but severe neurological syndrome characterized, in its classic form, by the acute onset of ocular disturbances, ataxia, and cognitive impairment. It is caused by a deficiency of thiamine (vitamin B1) and mainly affects chronic alcoholics, although it can also affect patients with pathologies that lead to malnutrition. We present a case of a 58-year-old woman, who presented with significant weight loss over the past 6 months and who came to the emergency department for episodes of repetitive vomiting and a sleepy state. The patient underwent blood chemistry tests and a brain CT scan, which revealed symmetrical and bilateral hypodensity of the medial portion of the thalamus, tectal plate, and periaqueductal gray matte, suggestive of WE. She was subsequently referred to the Department of Neurology and underwent a brain MRI, which confirmed the clinical suspicion. She also had an abdominal CT scan and ileo-colonoscopy and was diagnosed with Crohn's disease. Immediately after the clinical diagnosis of WE, a replacement therapy based on intravenous thiamine at high doses was promptly set up, and the patient improved from a clinical point of view.

Wernicke encephalopathy can be difficult to diagnose when it occurs in non-alcoholic patients; WE associated with IBD is a rare condition, and it can present with atypical and more subtle symptoms. Radiologists and physicians must be aware of this condition and imaging findings for rapid diagnosis and treatment.

## Introduction

Wernicke encephalopathy (WE) is a neurological condition resulting from a thiamine (vitamin B1) deficiency. This syndrome was classically described as a clinical triad consisting of altered mental status (i.e., confusion or dementia), ataxia, and nystagmus (or ophthalmoplegia). However, about a third of patients present with this complete triad [1,2]. The most common condition associated with WE are chronic alcohol abuse, but it is also important to remember that there are nonalcoholic causes of thiamine deficiency [3]; these include individuals with malnutrition, schizophrenia, and anorexia nervosa. Other common causes of WE include terminal cancer, starvation, strict diets, inflammatory bowel disease, bowel obstruction, AIDS, breastfeeding without supplements, and systemic disorders like tuberculosis and uremia [4]. If Wernicke encephalopathy is not treated properly, patients develop Korsakoff syndrome, a form of anterograde and retrograde amnesia with confabulation and gait abnormalities [5], a condition that is often irreversible.

The diagnosis of WE can be made based on Caine's operational criteria [6]: the classic symptom triad, autopsy evidence, or a rapid response to thiamine treatment.

It is important to highlight that nonalcoholic patients who are affected by WE are likely to present with different clinical symptoms and imaging findings than those of patients with alcoholism [7,8]. Diagnosis of WE should be considered if a patient has altered food ingestion or absorption or unbalanced nutrition, even if the patient shows only 1 of the classic 3 symptoms. It is important to consider that one or more symptoms appear later in the disease [7].

Imaging is the most valuable method to confirm the diagnostic suspicion [7]. CT is often non-specific, appearing negative, especially in the acute phases of the disease; the evidence of areas of hypoattenuation in typical and atypical locations is appreciated in the subacute and chronic phases [9]. MRI has high specificity for diagnosing WE and typically shows abnormal T2/FLAIR intensity alteration in bilaterally symmetrical lesions in the thalami, mammillary bodies, tectal plate, and periaqueductal area [10].

Treatment is based on parenteral administration of thiamine. After thiamine treatment, the acute symptoms of encephalopathy improve within the first week, but they usually take 1-3 months to resolve [11]. Magnesium deficiency is frequent in WE, especially chronic WE, so magnesium supplementation must be initiated because it is a cofactor for many thiamine-dependent enzymes [12]. Generally, the global confusion state rapidly resolves with adequate thiamine replacement, but ophthalmoplegia and ataxia may continue [13].

Wernicke encephalopathy is a medical emergency requiring immediate emergent attention, although the onset of the disease may be acute or chronic [14].

## Case presentation

A 58-year-old Caucasian female, affected by cognitive impairment and hypertension, arrived at the Emergency Room due to episodes of food vomiting and sleepiness.

Here, she underwent blood tests, documenting marked anemia (Hb 6.6 g/dL), chest X-ray, and non-contrast brain CT scan.

Anamnestic findings have a long history of vomiting, progressive, and significant weight loss (about 50 kg in 6 months), and nutritional replacement therapy with multivitamin complexes on medical indication. For about a month, the patient's sister had been reporting episodes of transient alteration of consciousness, disorientation, inability to recognize familiar faces, and speech disorder with difficult-to-understand words. Furthermore, she has been unable to walk, maintain a sitting position, and is bedridden.

**Fig. 1:**
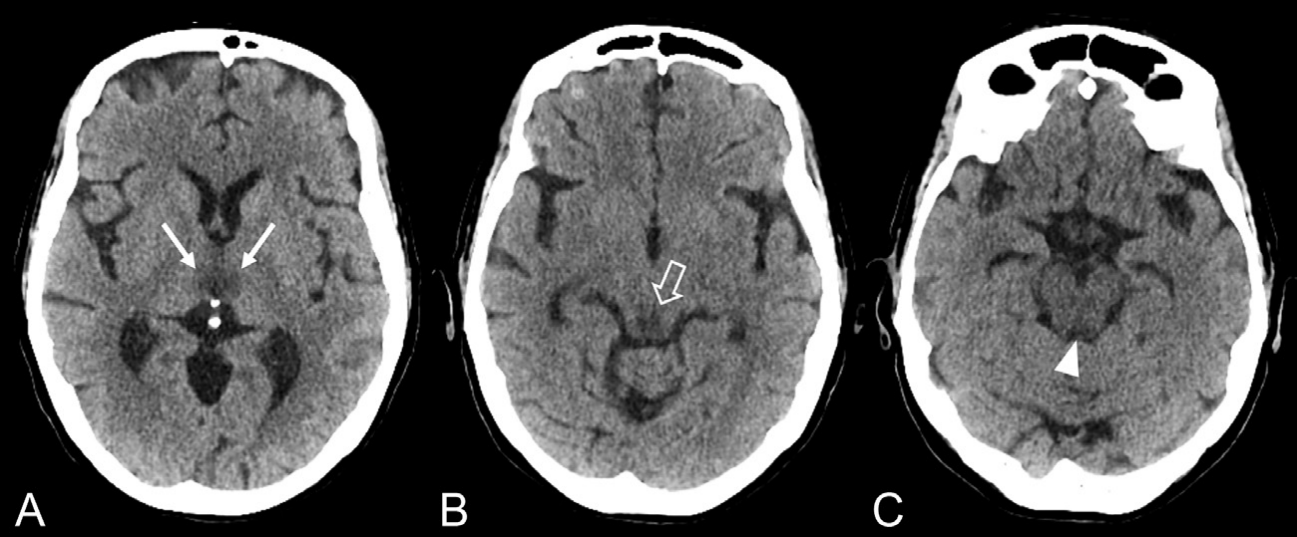
Unenhanced CT scan shows hypoattenuation in both medial thalami (thin arrow in A), the tectal plate (arrow in B) and periaqueductal region (arrowhead in C).

The brain-unenhanced CT scan showed the presence of symmetrical and bilateral hypodensity of the medial portion of the thalamus, tectal plate, and periaqueductal gray matter (Fig. 1).

In relation to the anamnestic information and the CT findings, the suspicion of Wernicke encephalopathy was raised. The patient was admitted to the neurology unit. The neurological objective examination on admission showed the patient alert but tending to drowsiness, not oriented in time and space, oriented to her person, ideomotor slowing, absent spontaneous speech, understanding, and execution of simple orders, upward gaze limitation with large upward nystagmus tremors, flexion posture of the upper limbs, hypoesthesia in the lower limbs, diffuse hypotrophy and hypotonia in the four limbs, weak osteotendinous reflexes in the four limbs, bilaterally mute hallux.

Sensitivity and cerebellar tests were not assessable. Furthermore, on general physical examination, the patient appears cachectic, undernourished, and dehydrated. In the immediate clinical suspicion of Wernicke's disease due to malabsorption, infusion therapy with high doses of thiamine was instituted.

**Fig. 2:**
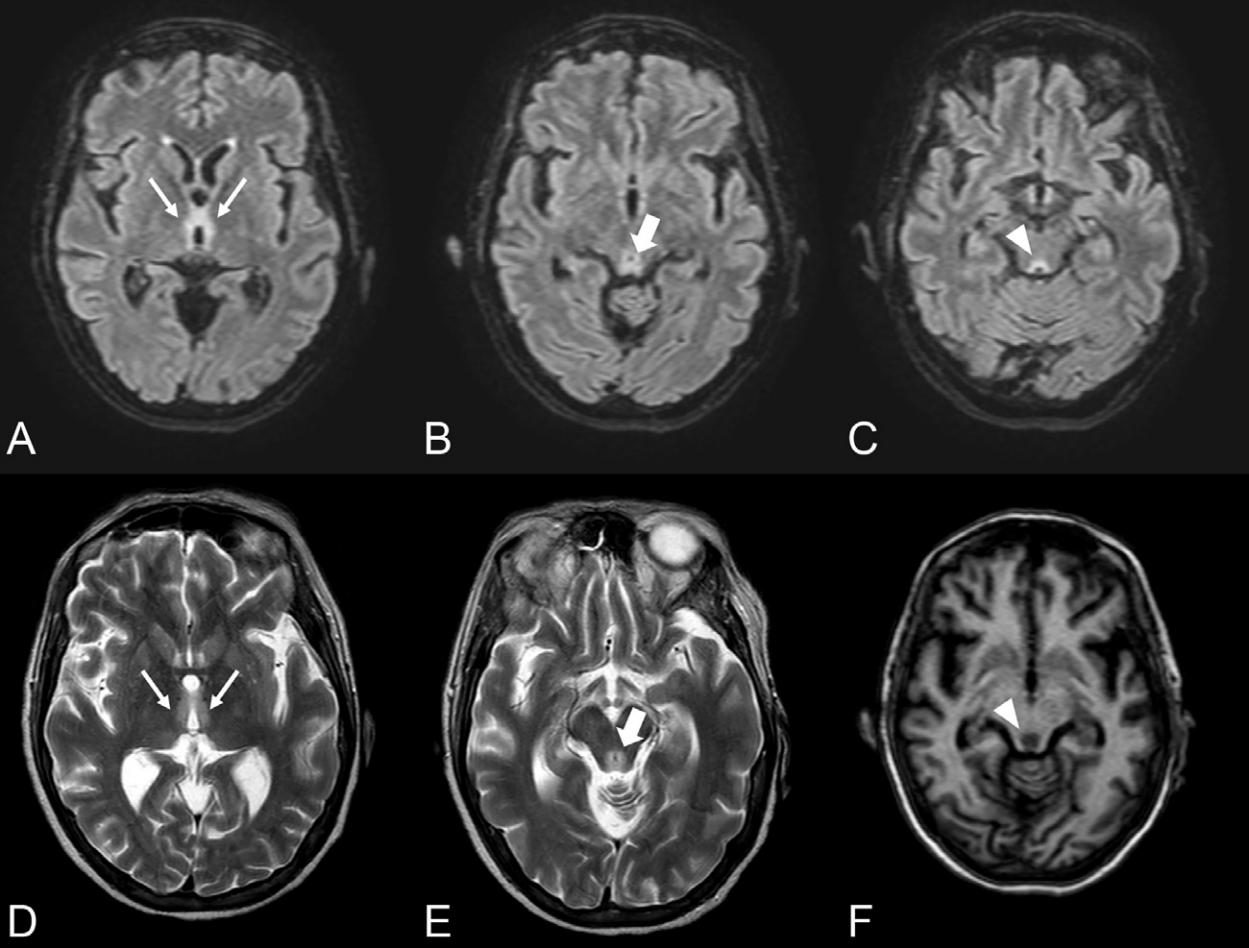
Axial fluid attenuated inversion recovery (FLAIR) (A,B,C), T2-weighted (D,E). High signal intensity is observed in both medial thalami (thin arrow in A and D), in the tectal plate (arrow in B and E) and in the periaqueductal region (arrowhead in C). T1-weighted image (F) shows low signal intensity in the periaqueductal region.

Brain MRI done 3 days after admission revealed a T2/FLAIR high signal area, symmetrical and bilateral, in the medial thalami, tectal plate, and periaqueductal grey matter (Fig. 2).

**Fig. 3:**
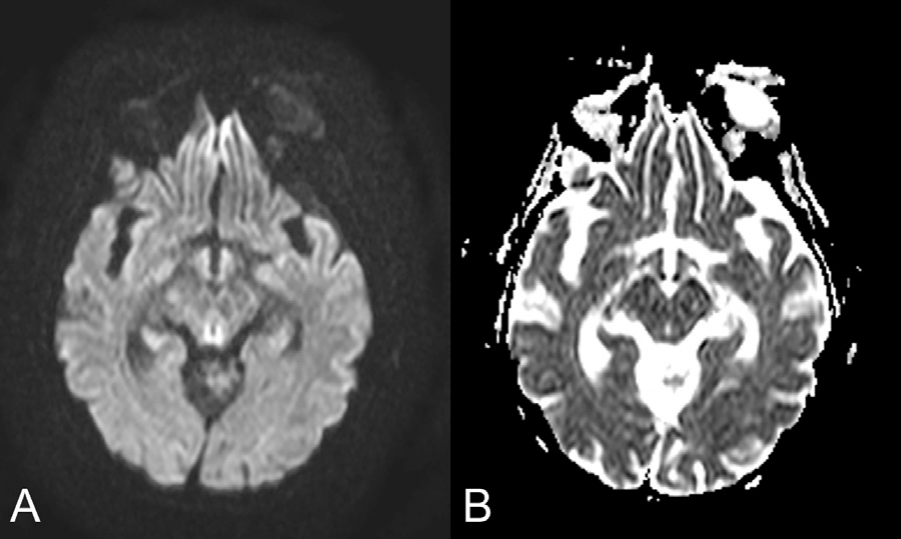
Axial diffusion-weighted imaging b-1000 (DWI) shows restricted diffusion, with high signal intensity in the tectal plate (A) and reduced intensity on ADC values (B).

Diffusion-weighted imaging shows restricted diffusion of these areas, with high signal on the b1000 image and low signal intensity on the apparent diffusion coefficient (ADC) map (Fig. 3).

**Fig. 4:**
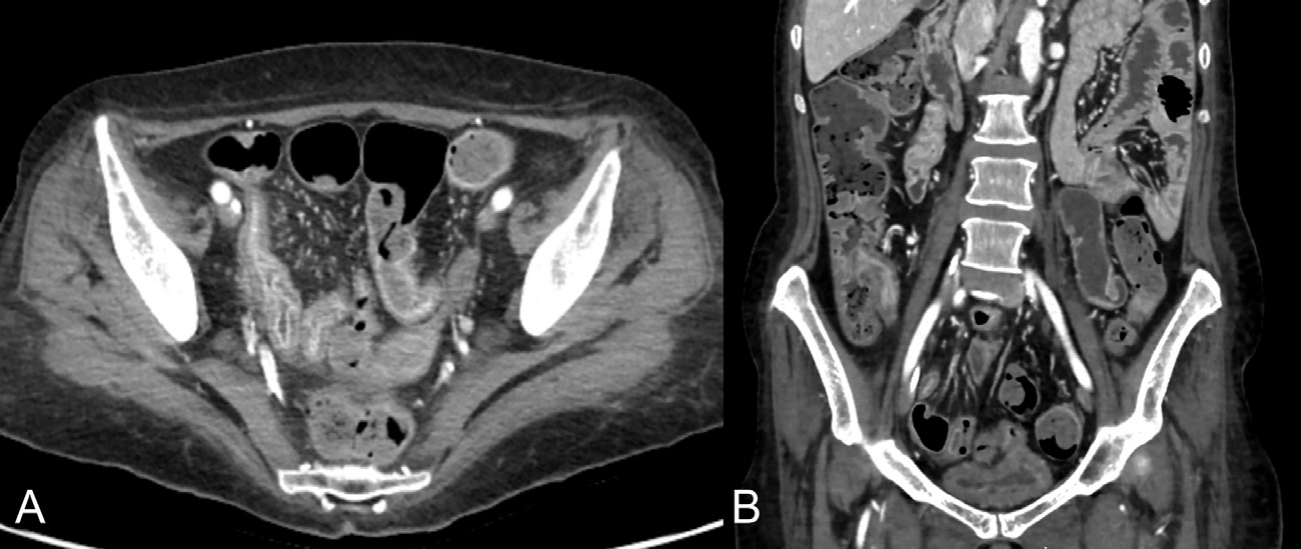
Axial (A) and coronal (B) contrast-enhanced CT images showed bowel wall thickening of the terminal ileum, with mural hyperenhancement and comb sign; these findings are suggestive of inflammatory bowel disease (IBD).

Due to severe anemia, the patient underwent a transfusion of concentrated red blood cells and underwent a contrast-enhanced CT scan of the abdomen to rule out acute bleeding; the CT scan showed bowel wall thickening of the terminal ileum, with mural hyperenhancement and comb sign, these findings are suggestive of inflammatory bowel disease (IBD) (Fig. 4).

In addition, she underwent an internist consultation, following which appropriate nutritional therapy, rehydration, antibiotic, and corticosteroid therapy for Crohn's disease, and subsequent indications for elective colonoscopy were set. Ileocolonoscopy with biopsy confirmed the diagnosis of Crohn's disease.

During the hospital stay, the patient underwent an improvement in her general clinical conditions. At the time of discharge, the neurological physical examination showed the patient to be alert, cooperative, oriented, speaking spontaneously, and responding to commands; upward gaze limitation was absent, and upward nystagmus jerks persisted.

## Discussion

Most cases of Wernicke's encephalopathy are seen in alcoholics, but it is essential to remember that numerous cases exist in which the disease is not related to alcohol [3].

Among the causes of non-alcoholic WE, inflammatory intestinal diseases (IBD) deserve mention, including Crohn's disease (CD) and ulcerative colitis (UC) [15,16]. In these pathologies, reduced intake of nutrients, malabsorption, vomiting, and side effects of drugs can cause significant nutritional deficiencies, including thiamine deficiency [16,17].

A recent systematic review of the literature showed that the correlation between WE and IBD is not frequent, with a prevalence rate of 0.3% [17]; in particular, the association with Crohn's disease was found in 21 cases out of the 31 described in the literature.

Nausea and vomiting represent the primary causes of WE in CD and UC, especially in patients who become malnourished due to prolonged vomiting [17].

In the presented case, despite the absence of alcohol consumption and the presence of vague neurological symptoms, the suspicion of metabolic encephalopathy was related to the CT findings associated with the patient's history of vomiting and weight loss, as suggested by the analysis of a series of patients with non-alcoholic WE [18], in which 7 out of 8 patients presenting persistent vomiting, it is therefore likely that the recent continuous vomiting promoted the onset of the encephalopathy of our patient.

Non-alcoholic WE can also be caused by other clinical conditions like gastrointestinal surgery, gastrointestinal tract diseases, pancreatitis, psychiatric diseases, and other risk factors that can lead to dietary deficiency and gastrointestinal absorption disorders [19].

Thiamine is converted in its biologically active form, thiamine pyrophosphate, in neuronal and glial cells. It is an essential coenzyme in many biochemical pathways in the brain [20]. Thiamine deficiency leads to brain lesions, visible on neuroimaging, in more susceptible areas of the brain with high thiamine content, such as the medial thalamus and periventricular region of the third ventricle, periaqueductal area, mammillary bodies, and midbrain tectum, that are considered “typical” sites of involvement [21]; these regions are more affected by alcoholic patients.

In contrast, in non-alcoholic patients, the involvement of “atypical” sites is more frequent (Table 1), in association with typical ones [8,10].

**Table 1:** Typical and atypical sites of involvement of WE [9].

Typical locations	Atypical locations
- Thalami - Mammillary bodies - Tectal plate - Periaqueductal area	- Putamen - Caudate - Splenium of the corpus callosum - Dorsal medulla - Pons - Red nucleus - Substantia nigra of the midbrain - Cranial nerve nucleus - Vermis - Dentate nucleus - Paravermian region of the cerebellum - Fornix and pre- and postcentral gyri

Suggestive findings on brain imaging of WE include bilateral and symmetric areas of altered density on CT or signal intensity on MRI, located in typical or atypical locations [9]; even though, in case of negative imaging, the diagnosis cannot be excluded [22,23]

CT has lower sensitivity, especially in the acute phase of the disease; it can identify areas of hypodensity in typical or atypical locations in the subacute or chronic phases and can easily recognize the hemorrhagic forms [24].

The case we have described underlines the importance of CT imaging, even in an emergency setting; identifying areas of hypodensity in typical locations has allowed, in association with an accurate medical history, to raise the suspicion of disease.

MRI represents the method of choice for confirming the diagnosis; the findings consist of areas of signal hyperintensity in T2-weighted and fluid-attenuated inversion-recovery (FLAIR) sequences in typical or atypical locations [25]. The use of a paramagnetic contrast medium is indicated in suspected WE; the areas that present contrast enhancement reflect the breakdown of the blood-brain barrier and correspond to lesions in the acute phase [23].

The role of diffusion-weighted sequences (DWI) and apparent diffusion coefficient (ADC) is unclear [26] due to the simultaneous presence of areas of restricted, normal, or increased diffusion [26]; in particular, the areas of diffusion restriction reflect the presence of cytotoxic edema of neurons or astrocytes and those with normal or increased ADC values represent areas of vasogenic edema; in a retrospective study [27] restricted diffusion was seen in 100% of non-alcoholic and 66% alcoholic WE patients although the sample examined in this study was not large.

In our case, the MRI findings were in a typical location (periaqueductal gray matter, tectal plate, and medial thalami) and there was a restriction in DWI.

As reported by Oudman et al. [17], MRI is very sensitive in identifying areas of altered signal in IBD - WE, despite a low sensitivity (53%) in patients with alcohol-related WE; this consideration could reflect a delay in the identification of WE or a worse outcome in the group of patients with CD or UC [17].

## Conclusion

Wernicke encephalopathy can be difficult to diagnose when it occurs in non-alcoholic patients; WE associated with IBD is a rare condition, and it can present with atypical and more subtle symptoms. Since this pathology, if not recognized and consequently not treated, can lead to death, it is essential for radiologists and clinicians to be aware of this condition and to suggest this clinical suspicion even in patients who present vague neurological symptoms and who have a condition of malnutrition or malabsorption.

## Data availability statement

The data presented in this study are available on request from the corresponding author.

## Ethical approval

Not applicable. The study was conducted according to the tenets of the Declaration of Helsinki. This was a case report without any type of experimental intervention, and ethical approval was therefore waived.

## Patient consent

Written informed consent for this case report was obtained from the patient.
